# Characteristics of Frequent Attenders Compared with Non-Frequent Attenders in Primary Care: Study of Remote Communities in Taiwan

**DOI:** 10.3390/healthcare8020096

**Published:** 2020-04-13

**Authors:** Shih-Chao Kang, Chun-Chi Lin, Chia-Chen Tsai, Yin-Han Lu, Chun-Feng Huang, Yu-Chun Chen

**Affiliations:** 1 Division of Family Medicine, National Yang-Ming University Hospital, Yilan 260, Taiwan; insly58@hotmail.com (Y.-H.L.); duz555@yahoo.com.tw (C.-F.H.); 2 Faculty of Medicine, School of Medicine, National Yang-Ming University, Taipei 112, Taiwan; yuchn.chen@gmail.com; 3 Division of Occupational Medicine, Taipei Veterans Hospital, Taipei 112, Taiwan; doc1554j@gmail.com; 4 Department of Internal Medicine, National Yang-Ming University Hospital, Yilan 260, Taiwan; jjthsai@yahoo.com.tw; 5 Department of Family Medicine, Taipei Veterans Hospital, Taipei 112, Taiwan

**Keywords:** community medicine, family medicine, frequent attendance, ICD-10 code, primary care, Taiwan

## Abstract

Frequent attenders (FAs) have an impact on the medical resources and the quality of care. In Taiwan, the characteristics of FAs remain unclear in primary care. Outpatient data were screened from a central clinic within six surrounding fishing villages in northeastern Taiwan in 2017. FAs were defined as those who made >18 visits in one year, and those who made ≤18 visits were defined as non-frequent attenders (NFAs). Data of FAs and NFAs were collected and compared. The major diagnoses were reported using International Classifications of Diseases, Tenth Edition (ICD-10) codes. A total of 1586 subjects and 9077 visits were enrolled, including 119 FAs and 1467 NFAs. FAs had a more advanced age compared to NFAs. Both FAs and NFAs had more visits in summer. FAs had consumed high prescriptions and related costs. FAs also had higher therapeutic and first visit costs than NFAs. Comparing with age- sex-matched NFAs, FAs were positively associated with musculoskeletal diseases (M00-M99), hematological diseases (D50-D89), endocrine diseases (E00-E90), and mental disorders (F00-F99). Large-scale local datasets and suitable definitions of FAs for Taiwanese subjects are needed.

## 1. Introduction

Frequent attenders (FAs) who utilize a number of medical resources with limited therapeutic effect are not rare in outpatient services. In Western countries that have public doctors and referral systems, this phenomenon has been widely discussed due to its impact on the distribution of medical resources and quality of care. Previous studies in Europe and the United Kingdom have reported certain risk factors and characteristics for FAs, including female predominance, psychological/somatoform disorders, chronic obstructive pulmonary diseases, diabetes mellitus, and cancer [1,2,3,4].

In Taiwan, the National Health Insurance (NHI) program since 1995 encouraged the expansion of private medical services, including hospitals and primary clinics. Currently, the NHI program in Taiwan covers 99% of the population and provides easily accessible medical care, short waiting times, relatively low health care costs, and affordable insurance fees [5,6]. Due to the large number of primary clinics and fee-for-service payment with low out-of-pocket fees, Taiwan has accessible primary care services, and patients make a higher mean number of visits compared with Western countries. In 2017, Taiwanese residents utilizing primary care made a mean of 11.4 visits per year, and following the addition of hospitals-affiliated outpatient services this has increased to 15.4 visits per year [7]. Comparing with members of Organization for Economic Co-operation and Development, the mean visits per year of Taiwan were listed among the top three countries, just second to South Korea and Japan [8,9]. However, differences in the quality and distance between urban and rural health care services remain inevitable [10]. Due to limited medical resources and countrywide budgets, the existence and increasing number of FAs are expected to impact the quality of care and overall utilization of medical care. At present in Taiwan, there are no relevant studies on FAs and their overall impact. The existence of FAs is inevitable; however, their characteristics in Taiwan remain unclear.

In contrast to urban or suburban areas, the importance of primary care was more significant in remote areas. Based on the health care experiences and accumulated data of managing a university hospital-affiliated clinic in a remote area of northeastern Taiwan, the aim of the current study was to investigate the characteristics of FAs compared with non-frequent attenders (NFAs) in primary care of remote communities.

## 2. Materials and Methods

### 2.1. Study Population, Setting, and Design

**Figure 1:**
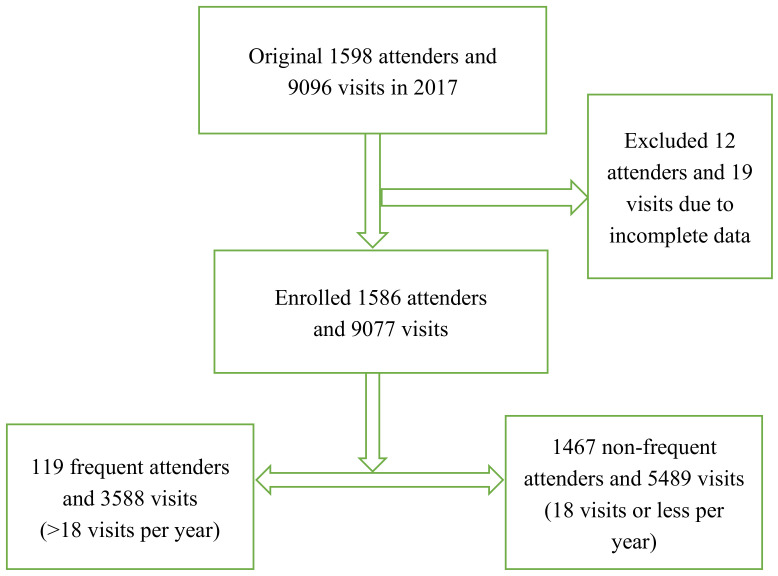
The design of the study.

Descriptive and retrospective analyses were performed. Outpatient data were obtained from a central primary care clinic on the northeast coast of Taiwan in 2017 which served six fishing villages. The clinic and its background communities were located in a remote area, 30 km from a major regional hospital. Having been affiliated with the major regional hospital from 2014–2018, the clinic provided primary care, undergraduate medical education, and research data. The population of the six fishing villages was 4816, estimated in February 2018 [11]. Patients with incomplete data including age or sex were excluded from the study. The study design, and inclusive and exclusive criteria are shown in Figure 1.

### 2.2. Definitions of Parameters

There is currently no standard definition of a FA. In Western countries, FAs are often defined as those accounting for the top 10% of all visits, or those who visit over 4 times per year [12,13,14]. In this study, FAs were defined as individuals who made >18 visits in 2017, and NFAs were defined as those who made ≤18 visits in 2017. We defined FAs as those who made >18 visits per year according to the reported average number of visits by the Bureau of NHI [7], the number of subjects, and correlations of logistic regression. It could achieve enough counts of a research group and presented significant differences between FAs and NFAs while the cut point was set at 18 visits per year.

The seasons were defined as follows: Spring (March to May), summer (June to August), autumn (September to November), and winter (December to February). From previous studies, seasonality could affect frequency of visits with some acute illness or complications of chronic illness, such as respiratory disease, trauma, cardiovascular diseases, and diabetes mellitus. However, it was still unclear if seasonality could affect FAs, and this study also investigated the issue. The medical costs were calculated automatically by in-clinic information software for claims data of the NHI program. According to the regulations of the Bureau of NHI, the definition of cost for diagnosis was payment of each visit for physicians, and the price differed by the level of medical institution and acute or chronic illness. In this study, cost for prescriptions refers to medications paid for by the NHI program. In addition, cost for therapeutics refers to non-medication services such as wound care and injections paid for by the NHI program. Cost for the first visit refers to the cost for the diagnosis at the subjects’ first attending visit, which costs more than subsequent fees for diagnoses.

The major diagnoses from each subject’s visit were defined according to The Tenth Revision of the International Statistical Classification of Diseases and Related Health Problems (ICD-10) codes. As well as general information on clinic visits, the outpatient data included an officially regulated adult preventive health survey, cancer screening, and smoking cessation services, which were classified as ICD-10 Z00-Z99 [15]. Due to being located in a remote area without pharmacists’ practices, the dispensing of prescriptions was performed by physicians at the setting clinic.

This study was approved by the Institutional Review Board of National Yang-Ming University Hospital (approval no. 2019 A016). The false hypothesis of this study was that FAs and age- and sex-matched NFAs had similar characteristics in community-based primary care.

### 2.3. Statistical Analysis

Data were obtained by searching the Health Information System for Clinics (Vision Asia Tech. Ltd., Taipei, Taiwan), and expressed as the mean ± standard deviation (SD) or percentage (%). All statistical analyses were performed using SPSS software (IBM SPSS version 22.0, SPSS Inc., Chicago, IL, USA). An independent *t*-test, chi-squared test, Fisher’s exact test, Pearson’s chi-squared test, and multivariate logistic regression analysis were performed. The correlated factors were adjusted by age and sex and presented as risk ratios (RRs), which were calculated with multivariate logistic regression analysis. A *p*-value < 0.05 was considered to indicate a statistically significant difference.

## 3. Results

### 3.1. Demographic Data

In 2017, a total of 9097 visits made by 1586 subjects met the inclusion and exclusion criteria and were enrolled in this study; this included 119 FAs and 1467 NFAs. Among them, 568 subjects (35.8%) made one visit in 2017. Table 1 shows the demographic data of all the included patients. Compared with the NFAs, the FAs had a more advanced age and more were aged over 40 years.

### 3.2. Seasonality and Medical Costs

The seasonal effect and medical costs are shown in Table 2. Both FAs and NFAs made more visits in the summer, and there was no significant difference between the two groups. In contrast to the NFAs, the FAs received a significantly higher number of prescriptions and averaged a higher cost of prescription per visit. The therapeutic and first visit costs of the FAs were also significantly higher than those of the NFAs.

### 3.3. Major Diagnoses of the Subjects’ Visits

The major diagnoses as reported by ICD-10 codes are listed in Table 3. The top five major diagnoses of the FAs were diseases of the musculoskeletal system and connective tissue (M00-M99); diseases of the respiratory system (J00-J99); diseases of the cardiovascular system (I00-I99); endocrine, nutritional, and metabolic diseases (E00-E90); and symptoms, signs, and abnormal clinical and laboratory findings, not elsewhere classified (R00-R99). The top five diagnoses of the NFAs were diseases of the respiratory system (J00-J99); diseases of the musculoskeletal system and connective tissue (M00-M99); diseases of the cardiovascular system (I00-I99); injury, poisoning, and certain other consequences of external causes (S00-T98); and diseases of the digestive system (K00-K93). The distributions of major diagnoses differed significantly between FAs and NFAs (*p* < 0.001 by Pearson’s chi-square test).

### 3.4. Factors Correlated with the FAs

The significance of various factors between the FAs and age- and sex-matched NFAs are shown in Table 4. After multivariate logistic regression analysis with adjustments for age and sex, the FAs were not correlated with seasonality. Compared with NFAs, the FAs were positively associated with musculoskeletal diseases (M00-M99), hematological diseases (D50-D89, most diagnosed as anemia), endocrine diseases (E00-E90, most diagnosed as diabetes mellitus), and mental disorders (F00-F99). In contrast, FAs correlated negatively with infectious diseases (A00-B99), respiratory diseases (J00-J99), skin diseases (L00-L99), trauma (S00-T98), health surveys or consults (Z00-Z99), and ear diseases (H60-H95).

## 4. Discussion

In this study, the demographic data revealed that the FAs had a more advanced age and a higher ratio of those over 40 years of age than the NFAs. In addition, more of the FAs were female (50.4% of the FAs versus 42.5% of the NFAs), although the difference was not statistically significant. Previous studies in Norway and Germany reported that FAs had a two-peak age distribution [1,16]. The current study showed that only 5.8% of the FAs were aged ≤40 years, and that most were >40 years. This is consistent with a report by the Tou-Cheng Township Household Registration Office, Yilan County, in 2018 that 16.8% of the population was over 65 years of age [17]. For the background communities in this study, the migration of the population and the number of newborns were minimal [11]. In aged communities where there is little migration of the population and few newborns, FAs are more likely to be older. The female predominance in the present study was similar to previous studies in Finland, although it did not achieve statistical significance [18,19]. As mentioned in the Methods, there is currently no standard definition of a FA. If the FAs were defined strictly (top 10% of all visits), the number of subjects would have been extremely low (*n* = 17). In addition, if the FAs were defined in wider terms (those visited >4 times per year), the differences between the FAs and NFAs would have been insignificant. This is also a limitation of a single-clinic study. Studies with a larger sample and multiple clinics are needed to clarify our results.

In this study, both the FAs and NFAs made more visits in the summer, and there was no significant difference between the two groups in seasonality. This may be due to the ratio of common diagnoses, as both the FAs and NFAs had common top three diagnoses (ICD J00-J99, M00-M99, and I00-I99), which accounted for >50% of the visits of the FAs and NFAs, respectively (51.1% versus 56.2%). Further logistic regression analysis also proved the noncorrelation of seasonality between the FAs and age- and sex-matched NFAs.

Analysis of medical costs and number of prescriptions revealed that the FAs consumed more resources and medical prescriptions, including increased costs and more days of prescription. In addition, compared to the NFAs, the therapeutic costs were higher in the FAs. This might reflect the frequent utilizations of therapeutic courses, such as wound care and intravenous/intramuscular injections in FAs. The cause of higher first visit costs in FAs remained unclear and requires multi-year studies to clarify it. Similar findings regarding the cost of prescriptions for FAs have also been reported in Poland and Finland [2,20].

In this study, the distribution of major diagnoses differed significantly between the two groups. Of the top five diagnoses, endocrine diseases (E00-E90) and medically unexplained symptoms (R00-R99) were common in the FAs, compared to trauma (S00-T98) and digestive diseases (K00-K93) in the NFAs. These findings reflect specific characteristics of FAs, including chronic metabolic/nutritional diseases (e.g., diabetes mellitus, thyroid abnormality, or malnutrition), and somatoform disorders, as shown by R00-R99. In contrast, the significance of S00-T98 and K00-K93 in the NFAs also reflected the effect of mean age, which was relatively younger and in keeping with the working age. It is known that medically unexplained symptoms (R00-R99) are correlated with somatoform disorders, anxiety, and depression, which are classified as F00-F99 [21,22]. The number of actual visits for psychological or mental disorders may be underestimated in FAs, and further investigations for medically unexplained symptoms are warranted.

We identified several interesting issues via multivariable logistic regression analyses. In contrast to the age- and sex-matched NFAs, certain positive correlations of the FAs became more significant, such as musculoskeletal diseases (M00-M99), hematological diseases (D50-D89), endocrine diseases (E00-E90), and mental disorders (F00-F99). In contrast, the major diagnoses that correlated negatively with FAs were acute illness mostly (A00-B99, J00-J99, S00-T99, S00-T98, and H60-H95) and periodic health survey (Z00-Z99). Such results reflected that some chronic illnesses may cause frequent attendance, and FAs paid less attention to consults of health behaviors and periodic health survey. These results revealed that FAs in primary care have unique problems. Unique major diagnoses for FAs have also been reported in studies conducted in the West [1,3,4,16,23]. Further studies are warranted to investigate the aforementioned unique and health problems of FAs, and also to help guide clinical practice to improve the quality of primary care for FAs. Due to the limited availability of multi-year claims’ data, the actual number of outpatients with a first diagnosis could not be calculated in this study. Multi-year data analyses for this issue could be considered in future studies.

The definition of FAs >18 visits per year and high mean outpatient visits [8,9] in Taiwan had implied many things, such as low insurance fees, accessible health services, wide insurance payment of medical services and prescriptions, scarcity of strictly referral systems, and other factors. For improving such phenomenon, NHI had supported primary clinics to organize family physicians’ medical groups and to join home medical care for community-oriented primary care. For referral systems, NHI had also performed reformations such as adjustments of payment and electronic referrals to encourage the cooperation between primary clinics and hospitals. The aforementioned reformations require time to observe the effects.

There are several limitations to the current study. The number of subjects was limited due to the study being conducted and the data were extracted from a single clinic, although it was a typical central health care center for surrounding remote communities. Among NFAs, it is possible that some of them may visit hospitals. However, distance is a limitation of attendance for community-dwelling elderly, especially those who live in rural areas. For decreasing such confounding factors, setting a clinic in remote communities (30 km to nearby major hospital) and cloud database checkup were conducted in this study, but the limitation was inevitable. In addition, comorbidities were not included due to limitations with the claims’ data. Some items, such as occupation, marital status, obesity, lifestyle, and history of cigarette or alcohol use, were lacking due to incomplete chart records. The scarcity of the aforementioned items would also affect the completeness of regression analyses and be confounding factors. Due to the study setting of a rural population, urban-rural comparison was indicated for the future. The medical services and diagnostic tools of the primary care clinic were limited, and this may have affected the accuracy of the major diagnoses, especially those listed as ICD R00-R99.

## 5. Conclusions

As mentioned in the introduction, this study investigated the characteristics of FAs compared with non-frequent attenders (NFAs) in primary care of remote communities. Compared with the age- and sex-matched NFAs, the positive associations with M00-M99, D50-D89, E00-E90, and F00-F99 indicated that the FAs often had musoskeletal, psychological, hematological, and metabolic/endocrine problems. The FAs also had an increased number of days on prescriptions. Both FAs and NFAs visited mostly in the summer, and their visits did not differ significantly. From the current study, we could realize unique health problems of FAs in remote primary care, and provided references and improvements of health care policies for remote regions. Local datasets for FAs in Taiwan have yet to be established. The current study is the first approach for FAs in primary care in Taiwan, and further studies are required to standardize the definition of a FA in Taiwan. Multiple-year or multiple-center studies of community health problems should be performed in follow-up studies, and these results should guide clinical practice to improve the quality of primary care for FAs.

## Figures and Tables

**Table 1:** Demographic data for all subjects (*n* = 1586).

Characteristic	FAs * (*n* = 119)	NFAs (*n* = 1467)	*p*-Value
Mean age (y/o)	65.60 ± 16.20	42.40 ± 21.24	<0.001 ^a^
Mean visits per year (times)	30.18 ± 13.41	3.74 ± 3.97	<0.001 ^a^
Sex (male/female)	59/60	843/624	0.058 ^b^
Age distribution (%)			<0.001 ^b^
<20 years old	1 (0.8)	245 (16.7)	
21–40 years old	6 (5.0)	473 (32.2)	
41–65 years old	53 (44.5)	544 (37.1)	
>65 years old	59 (49.6)	205 (14.0)	

* FAs, frequent attenders; NFAs, non-frequent attenders. ^a^ Independent *t* test. ^b^ Chi-square test.

**Table 2:** Season and cost of subjects’ visits (*n* = 9077).

Parameters ^d^	FAs * (*n* = 3588)	NFAs (*n* = 5489)	*p*-Value
Season of visits (%)			0.328 ^a^
Spring (March to May)	927 (25.8)	1438 (26.2)	
Summer (June to August)	943 (26.3)	1458 (26.6)	
Autumn (September to November)	893 (24.9)	1277 (23.3)	
Winter (December to February)	825 (23.0)	1316 (24.0)	
Prescription count (days)	10.59 ± 11.63	6.41 ± 9.08	<0.001 ^b^
Mean cost per visit (TWD ^c^)			
Cost of diagnosis	223.48 ± 152.08	227.61 ± 119.01	0.148 ^b^
Cost of prescription	211.53 ± 368.21	129.76 ± 258.38	<0.001 ^b^
Cost of therapeutics	76.96 ± 231.05	65.58 ± 264.64	0.035 ^b^
Cost of first visit	66.56 ± 229.33	42.12 ± 183.33	<0.001 ^b^

* FAs, frequent attenders; NFAs, non-frequent attenders. ^a^ Chi-square test. ^b^ Independent t test. ^c^ TWD, New Taiwan Dollar. ^d^ The definitions of parameters were seen in Section 2.2 of Materials and Methods.

**Table 3:** List of the major diagnoses from subjects’ visits ^a^.

Classification of ICD-10 Codes	FAs * n = 3588(100%)	NFAs *n* = 5489(100%)
A00-B99 Infectious and parasite diseases	26 (0.7)	80 (1.5)
J00-J99 Diseases of the respiratory system	613 (17.1)	1942 (35.4)
K00-K93 Diseases of the digestive system	281 (7.8)	434 (7.9)
L00-L99 Diseases of the skin and subcutaneous tissue	202 (5.6)	403 (7.3)
M00-M99 Diseases of the musculoskeletal system and connective tissue	619 (17.3)	586 (10.7)
N00-N99 Diseases of the genitourinary system	94 (2.6)	104 (1.9)
O00-O99 Pregnancy, childbirth and the puerperium	1 (0.03)	0 (0)
R00-R99 Symptoms, signs and abnormal clinical and laboratory findings, not elsewhere classified	289 (8.1)	313 (5.7)
S00-T98 Injury, poisoning and certain other consequences of external causes	136 (3.8)	464 (8.5)
C00-D48 Neoplasms	0 (0)	2 (0.04)
V01-Y98 External causes of morbidity and mortality	0 (0)	3 (0.05)
Z00-Z99 Factors influencing health status and contact with health services	58 (1.6)	165 (3.0)
D50-D89 Diseases of the blood and blood-forming organs and certain disorders involving the immune mechanism	22 (0.6)	11 (0.2)
E00-E90 Endocrine, nutritional and metabolic diseases	373 (10.4)	178 (3.2)
F00-F99 Mental and behavioral disorders	143 (4.0)	59 (1.1)
G00-G99 Diseases of the nervous system	46 (1.3)	57 (1.0)
H00-H59 Diseases of the eye and adnexa	18 (0.5)	36 (0.7)
H60-H95 Diseases of the ear and mastoid process	66 (1.8)	97 (1.8)
I00-I99 Diseases of the circulatory system	601 (16.8)	555 (10.1)

* FAs, frequent attenders; NFAs, non-frequent attenders; ^a^ *p* < 0.001 by Pearson’s chi-square test.

**Table 4:** Comparison of factors between FAs and NFAs under multivariate regression analyses.

Factors ^b^	Adjusted OR *	*p*-Value
Season of visits		
Spring (March to May)	1.024	0.718
Summer (June to August)	1.025	0.709
Autumn (September to November)	1.135	0.063
Winter (December to February)	-	-
Major diagnosis, ICD-10 codes ^a^		
A00-B99 Infectious and parasite diseases	0.526	0.011
J00-J99 Diseases of the respiratory system	0.576	<0.001
K00-K93 Diseases of the digestive system	0.943	0.573
L00-L99 Diseases of the skin and subcutaneous tissue	0.591	<0.001
M00-M99 Diseases of the musculoskeletal system and connective tissue	1.211	0.028
N00-N99 Diseases of the genitourinary system	0.850	0.322
R00-R99 Symptoms, signs and abnormal clinical and laboratory findings, not elsewhere classified	1.127	0.273
S00-T98 Injury, poisoning and certain otherconsequences of external causes	0.418	<0.001
Z00-Z99 Factors influencing health status andcontact with health services	0.439	<0.001
D50-D89 Diseases of the blood and blood-forming organs and certain disorders involving the immune mechanism	2.697	0.017
E00-E90 Endocrine, nutritional and metabolic diseases	2.305	<0.001
F00-F99 Mental and behavioral disorders	2.347	<0.001
G00-G99 Diseases of the nervous system	0.689	0.083
H00-H59 Diseases of the eye and adnexa	0.646	0.166
H60-H95 Diseases of the ear and mastoid process	0.628	0.010
I00-I99 Diseases of the circulatory system	-	-

* OR, odds ratio. ^a^ ICD-10, The Tenth Revision of the International Statistical Classification of Diseases and Related Health Problems. Reference value: NFAs. For ICD-10 codes, O00-O99(Pregnancy, childbirth and the puerperium), V01-V98(External causes of morbidity and mortality), and C00-D48 (Neoplasms) were excluded from logistic regression analysis. ^b^ Adjusted by age and sex.
